# Expanding the Toolbox: Utility of HistioTrak for Minimal Residual Monitoring in Pediatric Patients with Langerhans Cell Histiocytosis Treated with Targeted Therapy

**DOI:** 10.3390/cancers18081307

**Published:** 2026-04-20

**Authors:** Rainelle Nevers, Anusha Rajbhandari, Devon Roeming, Aly Anthony, Megan Gibbs, Anish K. Ray

**Affiliations:** 1 Department of Hematology and Oncology, Cook Children’s Medical Center, 1500 Cooper St 5th Floor, Fort Worth, TX 76104, USA; rainellenevers@my.unthsc.edu (R.N.);; 2 College of Pharmacy, University of North Texas Health Fort Worth, Fort Worth, TX 76107, USA; 3 Texas College of Osteopathic Medicine, University of North Texas Health Fort Worth, Fort Worth, TX 76107, USA

**Keywords:** Langerhans cell histiocytosis, BRAF V600E, MEK inhibitor, trametinib, HistioTrak

## Abstract

**Background/Objectives**: Langerhans cell histiocytosis (LCH) is a rare myeloid neoplasm characterized by the clonal proliferation of Langerhans-like dendritic cells and constitutive activation of the mitogen-activated protein kinase/extracellular signal-regulated kinase (MAPK-ERK) signaling pathway. Nearly 80% of ERK pathway activation can be attributed to B-Raf proto-oncogene, serine/threonine kinase (*BRAF* V600E), and mitogen-activated protein kinase kinase 1 (*MAP2K1*) variants, with *BRAF* V600E specifically detected in approximately 50% of pediatric LCH cases and associated with a higher risk of severe disease and treatment failure. The use of the HistioTrak clinical assay to detect the presence of *BRAF* V600E mutations in peripheral blood mononuclear cells (PBMCs) has emerged as a useful diagnostic tool and biomarker. **Methods**: This study is a single-center retrospective study that explores the favorable outcomes of treatment with trametinib on a small number of patients with LCH. We retrospectively analyzed the records of 11 children with LCH treated with trametinib at diagnosis as front-line therapy (*n* = 6), due to progressive disease (*n* = 3) or intolerance (*n* = 1) to chemotherapy, or at relapse (*n* = 1). **Results**: HistioTrak identified the presence of *BRAF* V600E PBMCs in five patients. In this small single-center retrospective cohort, trametinib was associated with favorable short-term outcomes in all patients, and serial HistioTrak testing appeared feasible in selected patients. **Conclusions**: Prospective studies are needed before routine diagnostic or monitoring use can be recommended.

## Simple Summary

The *BRAF* V600E mutation has been detected in approximately 50% of patients with Langerhans cell histiocytosis (LCH) and is associated with a higher risk of disease severity and treatment failure. HistioTrak clinical assay is a way to qualitatively detect the *BRAF* V600E mutation early in disease, allowing for early direction of management. The aim of this small single-center retrospective study was to evaluate the utility of HistioTrak for disease monitoring and describe clinical outcomes in pediatric patients with *BRAF* V600E-driven LCH treated with trametinib.

## 1. Introduction

Langerhans cell histiocytosis (LCH) is a rare clonal disorder of myeloid, Langerhans-like dendritic cells now recognized as a myeloid neoplasm. In children, its incidence is approximately five cases per million annually [1]. The pathological hallmark of LCH is the abnormal proliferation of CD1a^+^ and CD207^+^ dendritic cells within lesions. Clinically, LCH demonstrates striking heterogeneity, ranging from single-system involvement with isolated bone or skin lesions to multi-system disease involving multiple organs or diffuse dissemination. Multi-system LCH is further categorized as high-risk (risk-organ-positive; ROpos) or low-risk (risk-organ-negative; ROneg) based on the involvement of the liver, spleen, and/or bone marrow. This classification correlates with the risk of treatment failure and complications, such as diabetes insipidus and LCH-associated neurodegeneration (LCH-ND) [2].

Recent advances in the classification of histiocytic disorders have placed LCH within the Langerhans-related (L) subgroup, defined by recurrent somatic mutations in the mitogen-activated protein kinase–extracellular signal-regulated kinase (MAPK–ERK) pathway, particularly in B-Raf proto-oncogene, serine/threonine kinase (*BRAF*), and *MAP2K1* [3,4]. The most common alterations, *BRAF* V600E and *MAP2K1* mutations, account for up to 80% of cases. This molecular insight has substantial clinical implications, providing a rationale for MAPK-targeted therapies [5].

Treatment strategies are tailored to disease extent and the site of involvement. Local therapies can effectively control unifocal disease, whereas systemic therapy is indicated for multifocal or multi-system involvement. In pediatric patients, a combination of vinblastine and prednisone or single-agent cytarabine is commonly recommended as a first-line treatment. Oral agents such as methotrexate or hydroxyurea may be used in select cases, although responses are variable and monitoring can be challenging [6,7,8].

Advances in understanding the molecular pathogenesis of LCH have led to the integration of targeted therapies, particularly *BRAF* and MEK inhibitors, into the management of refractory cases [9,10,11]. *BRAF* inhibitors vemurafenib and dabrafenib, as well as the MEK inhibitor trametinib (brand name Mekinist), have demonstrated rapid and often durable responses in pediatric and adult cohorts [12,13,14,15,16,17,18], including a small group of pediatric patients at our institution [18]. However, one challenge that remains is the risk of relapse after discontinuation, which has been shown to frequently occur in adult patients treated with *BRAF* inhibitors. Other challenges include the unknown duration of optimal therapy, long-term tolerability, and the need to balance efficacy with minimizing cumulative toxicities, especially in children [11,12,13,14,15,16,17,18]. In our previous work, we have demonstrated favorable outcomes in pediatric patients with LCH treated with trametinib, including those initiated on trametinib at diagnosis as well as refractory cases [18].

With the advancement of targeted therapies and positive outcomes reported in recent case studies, there is an increasing emphasis on the early identification of patients harboring somatic mutations. Notably, the presence of the *BRAF* V600E mutation is associated with high-risk disease phenotypes, resistance to standard chemotherapy regimens, and an elevated risk of relapse [19]. This mutation is detected in approximately half of pediatric patients, with some estimates up to 60% [3]. The detection of *BRAF* V600E mutations plays a critical role in the diagnosis and management of systemic histiocytic disorders. Traditionally reliant on tissue biopsy, this process can now be complemented by cell-free DNA (cfDNA) analysis, which offers a noninvasive and reliable alternative for mutation detection and disease monitoring [20].

Monitoring minimal residual disease (MRD) using blood-based assays offers several advantages over traditional biopsy techniques. Conventional biopsy procedures, which often involve invasive methods such as needle aspiration or surgical excision, carry inherent risks, including infection, bleeding, exposure to anesthesia, and poor patient tolerance. In conditions such as LCH, where disease involvement is multifocal and spans multiple organ systems, biopsies may fail to capture active disease sites, potentially leading to an underestimation of disease burden. Moreover, longitudinal disease surveillance is challenging because repeated biopsies are impractical and burdensome. Classical molecular approaches for MRD detection are also limited by prolonged turnaround times, often requiring several days to weeks for results [21,22].

Circulating tumor DNA (ctDNA) has emerged as a valuable biomarker for noninvasive cancer detection and disease monitoring, providing real-time insights into tumor genomics and therapeutic response. ctDNA, which is shed into the bloodstream, often carries the same genetic alterations as the primary tumor, making it a highly sensitive and specific tool for assessing tumor burden [23]. Numerous studies have demonstrated its utility in tracking malignancies, such as anaplastic thyroid carcinoma, lung and colon carcinomas, and head and neck squamous cell carcinoma [24,25,26]. In the context of LCH, ctDNA reflects recurrent clonal mutations, including *BRAF* V600E, and can serve as a dynamic marker for monitoring disease progression and therapeutic response. When integrated with Next-Generation Sequencing (NGS), ctDNA analysis enables precision oncology by facilitating the identification of actionable mutations.

Droplet digital polymerase chain reaction (ddPCR) has also emerged as a particularly powerful method for detecting rare variants, such as *BRAF* V600E, with higher precision and sensitivity [27,28,29]. While ctDNA analysis relies on plasma free DNA, HistioTrak, a commercially available clinical assay through Cincinnati Children’s Hospital Division of Pathology, utilizes ddPCR to qualitatively detect the *BRAF* V600E mutation in peripheral blood mononuclear cells (PBMCs) with high sensitivity and specificity, even with a low disease burden [12,30]. The integration of HistioTrak offers a complementary blood-based approach to detecting mutation burden and monitoring disease over time.

A recent study by Lin et al. demonstrated that the presence of *BRAF* V600E-positive PBMCs at diagnosis is strongly correlated with systemic treatment failure and an increased risk of later development of LCH-ND [31].

Based on these emerging findings, we extended our previous work and further evaluated the relationship between *BRAF* V600E-positive PBMCs and treatment outcomes through an expanded retrospective analysis of pediatric patients treated with the targeted agent trametinib at our institution. We also focused on how HistioTrak testing may be integrated into clinical applications for diagnosis and treatment decisions in pediatric LCH.

## 2. Methods

This single-center, retrospective study evaluated children diagnosed with LCH who underwent assessment of circulating *BRAF* V600E mutation status and were treated with the MEK inhibitor, trametinib. Patients treated with trametinib for LCH and tested with HistioTrak were enrolled between May 2022 and September 2025 under protocols approved by the Institutional Review Board (IRB) at Cook Children’s Health Care System (IRB number: 2023-058; approved 26 September 2025), and the study was conducted in accordance with the Declaration of Helsinki. All patients provided written informed consent. Medical records from 11 patients were retrospectively reviewed, and all patient data was anonymized to maintain confidentiality.

Demographic, clinical, and treatment-related data were collected, including patient age, sex, disease extent according to classifications established by the Histiocyte Society, molecular findings based on available NGS sequencing results, prior treatments, and initiation, duration, and associated adverse effects of trametinib treatment. The cutoff date for these analyses was 31 October 2025.

HistioTrak is a commercially available ddPCR-based molecular assay that was used in this study to determine *BRAF* V600E status in patients undergoing treatment. Whole blood samples were collected and sent to Cincinnati Children’s Hospital Division of Pathology, Cincinnati, Ohio, USA. The analyte consisted of DNA extracted from PBMCs to result in highly sensitive and specific qualitative data capable of detecting low-level mutation signals consistent with minimal residual disease as described by Cincinnati Children’s [30]. HistioTrak was checked at least once in all patients. Molecular findings and the timing of the HistioTrak data were also collected. For some patients, HistioTrak and lesion sequencing were performed simultaneously at diagnosis and prior to initiation of trametinib. HistioTrak was collected in select cases during the course of treatment.

Trametinib was administered at the discretion of the treating clinical provider after an informed consent discussion with the family and according to institutional guidelines. Dosing was determined by body weight (starting dose of 0.015 mg/kg) and not escalated even as the patients continued to grow and increase in weight. Adverse events (AEs) were retrospectively identified and recorded based on clinician judgment noted in patient chart documentation. Supportive care interventions, including dose reduction to previously tolerated dose and decreased frequency of administration from daily to every two or three days as well as temporary treatment interruption, were implemented to manage AEs. In accordance with the manufacturer’s monitoring recommendations, baseline and annual cardiac and ophthalmological evaluations were conducted. All patients also underwent at least annual assessments with complete blood counts and blood chemistry panels. Only clinician-documented abnormalities were included in the analysis.

All patients were evaluated for response to treatment at monthly intervals for the first 2 months, and then, if clinically well, every 3 months (±15 days). Treatment response was assessed using clinical judgment supported by imaging studies, including X-ray, positron emission tomography/computed tomography (PET-CT), magnetic resonance imaging (MRI), and other modalities. Responses were categorized as no active disease, improving active disease, progressive active disease, or stable disease. Patients who underwent radiographic assessments both before and after trametinib initiation were classified according to the Response Evaluation Criteria in Solid Tumors (RECIST) version 1.1 [32].

## 3. Results

### 3.1. Overview of Patient Cohort

This study included 11 pediatric patients diagnosed with LCH between the ages of 0.3 and 9.5 years (median 1.5 years) (Table 1). Seven of these patients were male, and six were of Hispanic origin. Most patients (*n* = 7, 64%) presented with multi-system LCH (MS-LCH). Four patients had single-system LCH (SS-LCH), which involved either a single lesion (*n* = 2) or multifocal single-system involvement (*n* = 2). Patients with SS- and MS-LCH had lesions in the skin, bone, lymph nodes, or central nervous system (CNS). One patient with MS-LCH had special site involvement (Table 1).

The details of disease classification, lesion mutation, prior therapies, trametinib initiation, adverse effects, and responses at the last follow-up are summarized in Table 2. Genetic sequencing of the lesion was conducted in 10 patients, which identified the *BRAF* V600E mutation in seven, the *BRAF* p.N486_T491delinsK mutation in one, and *MAP2K1* alterations in two.

### 3.2. Clinical Outcomes

Three patients (#1, 5, and 11) had disease progression, one patient (#8) relapsed, and one patient (#10) had intolerance to adverse effects following prior treatments, which included cytarabine, methotrexate, vinblastine, and prednisone, as well as surgical interventions and topical steroids. This illustrates the diversity of traditional therapeutic approaches undertaken before initiating targeted agents.

Six patients (#2, 3, 4, 6, 7, and 9) initiated targeted therapy with trametinib at the time of diagnosis. The youngest patient (#2) began treatment with trametinib at 3 months of age. Patients 1 and 11 also started targeted treatment at less than a year of age after failing prior treatments. Notably, Patient 4 underwent surgical resection and initiation of targeted therapy at presentation.

Of the 11 patients, 10 had radiographically confirmed responses to trametinib, with normalization of PET avidity on PET-CT scans. This was typically within 1–4 months after initiating trametinib. One patient (#11) did not complete follow-up imaging after starting trametinib treatment and was lost to follow-up. All 10 remaining patients have remained relapse-free at the time of data cutoff; thus, they are considered to be in clinical remission with no evidence of active disease. Of note, Patient 10 completed a PET-CT scan at the time of trametinib initiation due to intolerance of initial chemotherapy treatment, which showed resolution of prior PET-avid lesions. None of the patients have required repeat imaging as they have remained in clinical remission at a median follow-up of 11 months (ranging from 1.5 months to 3.5 years).

Three patients (#2, 4, and 11) discontinued trametinib after 27, 26, and 12 months of treatment, respectively. Patients 2 and 4 discontinued treatment after achieving clinical disease remission and had a minimum of two planned years of targeted treatment. Patient 11 was lost to follow-up after one year.

While clinical outcomes have been positive in this cohort, adverse effects were reported in five patients (#1, 4, 5, 6, and 9). The most common adverse effect was rash (*n* = 3), with one patient experiencing improvement by decreasing frequency of trametinib administration. Three patients experienced diarrhea, constipation with an instance of hematochezia, and nausea. However, these adverse effects were mild and transient. None of these adverse effects required discontinuation of therapy. Notably, Patient 1 had a rash, recurrent infections, and a brief, self-limited episode of pancytopenia likely secondary to viral illness-induced transient marrow suppression. This patient was able to resume therapy at a reduced frequency while maintaining a good therapeutic response. Ultimately, no patient experienced dose-limiting toxicities or cardiac or ophthalmological dysfunction.

### 3.3. PBMC BRAF V600E Characteristics and Outcomes

A total of 11 patients underwent HistioTrak analysis. Among the seven patients with *BRAF* V600E-mutant tumors on NGS, three were HistioTrak-positive at diagnosis. Only one patient remained positive at a subsequent time point during disease progression while on oral methotrexate before transitioning to trametinib. Two patients were HistioTrak-positive at follow-up evaluations: one patient had multi-system LCH and progressed through cytarabine. The other patient had a solitary bone lesion and completed two years of trametinib therapy. This patient was HistioTrak-negative at treatment completion, converted to a positive status three months later, and subsequently reverted to a negative status without further therapy.

All other patients tested negative for HistioTrak during follow-up evaluations. One patient’s tumor was not sequenced; peripheral blood from this patient was negative for circulating *BRAF* V600E on HistioTrak.

## 4. Discussion

The management of LCH has changed with the recognition of key molecular drivers, especially activating mutations in the MAPK pathway, such as *BRAF* V600E [4,19]. Several case reports and reviews have illustrated the effectiveness of inhibiting the MAPK-ERK pathway, especially *BRAF* and MEK inhibition, in the treatment of LCH.

This retrospective study extends our previous work [18] and further demonstrates that trametinib is a safe and effective treatment for pediatric patients with LCH, regardless of disease extent or mutational status. All patients with single-system and multi-system LCH, including those with CNS involvement or CNS-risk lesions, responded well and achieved rapid remission, which was either radiographically confirmed on PET-CT or clinically evaluated as disease-free at short-term follow-up. Trametinib was overall well-tolerated. Two patients underwent adjustments to the dosing frequency without a decrease in efficacy. While the optimal duration of therapy is unknown, the general notion at our institution is to continue the targeted agent for at least 2 years and then closely monitor for signs of relapse after discontinuation. Two patients discontinued trametinib after a minimum of two years of treatment and successfully maintained remission without evidence of relapse as of October 2025, which is more than one year off targeted therapy.

While conventional monitoring relies on imaging and tissue biopsies, these approaches have inherent disadvantages, including invasiveness, sampling error, and delayed turnaround times. This highlights the need for real-time, minimally invasive biomarkers that can guide treatment and identify relapses earlier. Liquid biopsy approaches, particularly those involving circulating tumor DNA (ctDNA), may help address these gaps by tracking disease burden and treatment response without repeated procedures [33]. HistioTrak has emerged as a newer platform that uses ddPCR technology for ctDNA monitoring in histiocytic disorders, including LCH. In this study, we evaluated the performance of HistioTrak in clinical care, focusing on its feasibility, correlation with imaging and clinical assessments, and ability to monitor MRD in patients with LCH.

Given that circulating *BRAF* V600E is linked to higher-risk disease and resistance to standard chemotherapy, it would be beneficial for patients to have the HistioTrak test conducted early at the time of their LCH diagnosis. The turnaround time for the test is approximately 3–4 days, and it is relatively noninvasive compared to extensive tissue biopsies and imaging [1,19,34,35,36]. These traditional methods can be physically and psychologically challenging for younger patients and can place a significant emotional and logistical burden on families. They can also pose a logistical problem on study design as lesion genotyping typically requires several weeks to return.

In this study of 11 pediatric patients, five were identified as *BRAF* V600E-positive using the HistioTrak test, three at diagnosis, and two at subsequent follow-up evaluations. Among these patients, 40% (*n* = 2) showed resistance to traditional chemotherapy, leading to initiation of targeted therapy. Sixty percent (*n* = 3) had a more severe clinical phenotype, including two patients with CNS-risk lesion sites or CNS involvement of disease and one patient with severe multi-system disease with large bone and skin lesions causing functional impairment due to pathologic bone fracture, decreased range of motion, and extreme irritability and pain. Similarly, the two patients with confirmed *BRAF* V600E lesions but were *BRAF* V600E-negative on HistioTrak testing had either MS-LCH or unifocal SS-LCH. This includes one patient (#10), who would also be considered high-risk given CNS involvement and CNS-risk bone involvement. Conversely, all five patients with peripheral *BRAF* V600E presented at less than 2 years of age, while one patient who was negative for peripheral *BRAF* V600E presented at 9 years of age. Given the small sample size of this cohort, it is not possible to comment on whether the presence of peripheral *BRAF* V600E entirely correlates with a more severe clinical phenotype.

Additionally, patients with *BRAF* V600E-positive PBMCs did not necessarily experience worse outcomes than those who were *BRAF* V600E-negative. Eighty percent of peripheral *BRAF* V600E-positive patients were started on targeted therapy due to disease progression or relapse following prior treatments. One of these patients (#8) failed methotrexate and remained positive on HistioTrak test, prompting a transition to trametinib, leading to clinically and radiographically confirmed remission. Notably, the majority of the *BRAF* V600E-negative patients (83%) were started on targeted therapy at presentation upon an informed consent discussion with family; therefore, we cannot comment on any possible predicted outcomes that may have occurred if treated with standard-of-care chemotherapy protocols. It should be noted that Patient 10 was started on a targeted agent due to chemotherapy intolerance, but PET-CT at the initiation of trametinib had already shown clinical remission. Ultimately, all 11 patients achieved clinical remission at time of writing, regardless of peripheral *BRAF* V600E mutational status.

Furthermore, two of the five patients identified as *BRAF* V600E-positive using the HistioTrak test had low-level positives. These patients were started on trametinib due to disease progression after surgical interventions, which is different from other peripheral *BRAF* V600E-positive patients who had resistance to traditional chemotherapy. One patient demonstrated a low-level positive result only once, occurring three months after discontinuation of trametinib. The patient remained negative during treatment and at subsequent assessments conducted 6, 9, and 12 months after therapy cessation. At this time, there are no clinical guidelines for these results as their significance is not yet known, and it was decided to observe this patient only as they had completed two years of trametinib therapy. It is reassuring that they have also achieved remission for more than one year as of October 2025.

Utilizing ctDNA for minimal residual disease (MRD) monitoring provides an opportunity to track the effects of targeted therapy and chemotherapy long after treatment completion, offering insight into the durability of response. Serial assessment of *BRAF* V600E through liquid biopsy has been shown to correlate with disease activity and can detect molecular relapse earlier than imaging or clinical symptoms [23]. Continued monitoring of *BRAF* V600E in plasma or peripheral blood supports risk stratification of residual disease and helps distinguish relapse from remission. Some studies have suggested that *BRAF* V600E-positive peripheral blood mononuclear cells at diagnosis identify patients at the highest risk of treatment failure and neurodegeneration, and persistent or rising *BRAF* V600E levels during or after MAPK inhibition have been linked to relapse after treatment discontinuation [31,37,38]. However, our findings do not fully align with these previous studies, especially as our patients with peripheral *BRAF* V600E have been able to achieve clinical remission, including those who discontinued treatment. However, this study is limited by its retrospective nature, small sample size, and short follow-up period. We also recognize that there is limited understanding of the significance of positive or negative results when obtained at time points outside of new diagnosis or new relapse. In addition, the test currently only informs providers regarding the *BRAF* V600E mutation and not MAPK.

In the future, larger prospective clinical trials with more prolonged follow-ups in patients with LCH that incorporate peripheral *BRAF* V600E monitoring will be needed to further evaluate these findings. It may also be beneficial to develop more quantitative analyses for HistioTrak testing, which is currently only qualitative, to further assess the potential disease burden or as a strategy in treatment planning. This approach would enable clinicians to distinguish true remission from persistent subclinical disease, which is critical for risk stratification and timely intervention. By integrating *BRAF* V600E monitoring into routine follow-up, clinicians can make more informed decisions about therapy duration, escalation, or the need for targeted agents, ultimately personalizing care for patients with LCH.

## 5. Conclusions

This early institutional experience highlights successful treatment of pediatric LCH with a targeted agent, trametinib, including manageable toxicity and favorable remission rates at short-term follow-up. In addition, HistioTrak acted as a promising application of a less invasive biomarker for disease tracking that could be feasibly integrated into clinical applications. However, the retrospective nature and small sample size of this study limit the generalization of these results.

Ongoing efforts at this institution include more extensive biobanking and clinical-molecular annotation to support precision medicine strategies for pediatric LCH and related histiocytic disorders. Future prospective clinical trials with larger populations, especially given the heterogeneity of LCH, are also needed to further assess long-term outcomes of LCH treated with targeted agents like trametinib and how monitoring peripheral *BRAF* V600E can be incorporated into routine clinical care for better risk stratification and treatment planning.

## Figures and Tables

**Table 1:** Summary of patient demographics, disease classification, lesion site, and mutation status.

		*N* (%)
Sex		
	Male	7 (64%)
	Female	4 (36%)
Race/ethnicity		
	Hispanic or Latino	6 (55%)
	Non-Hispanic White	4 (36%)
	Non-Hispanic Black	1 (9%)
Age at diagnosis, median (range)		1.5 (0.3–9.5)
Disease classification		
	Single-system disease	4 (36%)
	Multi-system	7 (64%)
Lesion site		
	Skin	5 (45%)
	Bone	8 (73%)
	CNS involvement	2 (18%)
	CNS-risk	3 (27%)
	Special site	1 (9%)
PBMC *BRAF* V600E status prior to initiation of targeted therapy		
	Detected	3 (27%)
	Undetected	4 (36%)
	Unknown	4 (36%)
Age at inhibitor initiation, median (range)		1.8 (0.3–9.5)
Point of targeted therapy initiation		
	Presentation	6 (55%)
	Progression of disease/relapse	4 (36%)
	Chemotherapy intolerance	1 (9%)
PBMC *BRAF* V600E status after initiation of targeted therapy		
	Detected	3 (27%)
	Undetected	2 (18%)

**Table 2:** Clinical characteristics, treatment, outcomes, and HistioTrak testing of patients.

Patient Number	Age at Dx (Years)	Disease Classification/Site	Mutation Status	Prior Therapy	Age at Targeted Therapy Initiation (Years)	Years on Therapy	Current Therapy	Outcomes	Adverse Effects	Timepoint of HistioTrak Testing Diagnosis Subsequent	
1	0.3	MS-LCH Unifocal bone Axillary rash Oral lesions	*BRAF* V600E	Cytarabine	0.5	1.1	Trametinib	CR	Rash, recurrent infections, pancytopenia		Mid Therapy, positive
2	0.3	MS-LCH Skin Lymph node	N/A	N/A	0.3	2.3	None. Trametinib discontinued d/t disease remission	CR	N/A		Mid Therapy, negative
3	9.2	SS-LCH Unifocal Bone	*BRAF* V600E	N/A	9.2	0.4	Trametinib	CR	N/A	Negative	
4	1.8	SS-LCH Unifocal CNS-risk bone	*BRAF* V600E	Surgery	1.8	2.2	None. Trametinib discontinued d/t disease remission	CR	Rash		Mid Therapy—negative 3 mo off therapy—positive (low level) 6 mo off therapy—negative 9 mo off therapy—negative 12 mo off therapy—negative
5	0.5	MS-LCH Skin Lymph node	*BRAF* V600E	Topical steroids	1.1	1.2	Trametinib	CR	Diarrhea, constipation	Positive	
6	7.1	MS-LCH Unifocal bone Soft tissue Lymph node	*BRAF* p.N486_T491delinsK	N/A	7.1	0.5	Trametinib	CR	Nausea	Negative	
7	2	SS-LCH Multifocal Bone	*MAP2K1* F53_Q58>L F53_Q58>L	N/A	2	0.1	Trametinib	CR	N/A	Negative	
8	1.5	SS-LCH Skin, multi-site involvement	*BRAF* V600E	Methotrexate	1.8	0.1	Trametinib	CR	N/A	Positive	Mid Therapy—positive
9	9.5	MS-LCH Multifocal bone Lymph node	*MAP2K1* p.E102_I103del	N/A	9.5		Trametinib	CR	Rash, hematochezia	Negative	
10	1.1	MS-LCH Multifocal bone, including CNS-risk and special site lesions Calvarium Soft tissue, including CNS	*BRAF* V600E	Surgery Vinblastine and prednisone;	1.3	0.4	Trametinib	CR	N/A		Switch of therapy—negative
11	0.6	MS-LCH Bone, including CNS-risk lesions Calvarium Soft tissue, including CNS Lymph node	*BRAF* V600E	Surgery	0.7	1	None. Trametinib discontinued as lost to follow-up	NAD	N/A	Positive (low level)	

Abbreviations: CR, complete response (radiographically confirmed remission). MS, multi-system. N/A, not applicable. NAD, no active disease (clinically disease-free but no objective radiographic measures). SS, single system. Note: Mid Therapy refers to targeted treatment with trametinib.

## Data Availability

The original contributions presented in this study are included in the article. Further inquiries can be directed to the corresponding author.
